# Acute Pancreatitis and Disseminated Intravascular Coagulopathy in COVID-19 Infection: A Case Report

**DOI:** 10.7759/cureus.34104

**Published:** 2023-01-23

**Authors:** Mohammad Abu-Abaa, Ghassan Al-Qaysi, Ali Abdulsahib, Talar Acob

**Affiliations:** 1 Internal Medicine, Internal Medicine Residency Program, Capital Health Regional Medical Center, Trenton, USA; 2 Internal Medicine, Capital Health Regional Medical Center, Trenton, USA

**Keywords:** elevated lipase, bleeding, severe pancreatitis, disseminated intravascular coagulation (dic), covid 19

## Abstract

Coronavirus disease 2019 (COVID-19) is caused by severe acute respiratory syndrome coronavirus 2 (SARS-CoV-2) and can be a plausible trigger for both disseminated intravascular coagulopathy (DIC) and acute pancreatitis. We present an 85-year-old male patient who presented with altered mental status and tested positive for COVID-19 infection. He was hypoxic with an incremental need for oxygen. He had clinical as well as imaging evidence of acute pancreatitis. Clinical evidence of bleeding was noted and lab findings were suggestive of DIC. Despite aggressive initial management, his clinical status continued to deteriorate and comfort care was sought eventually. This case highlights COVID-19 infection as a possible trigger for acute pancreatitis as well as DIC. It also highlights some of the differences in COVID-19-induced DIC, which fulfills the diagnostic criteria of DIC but has atypical findings.

## Introduction

Around 80% of coronavirus disease 2019 (COVID-19) patients are asymptomatic while 20% progress to severe infection [1]. Coagulation abnormalities are common among COVID-19 patients with predominant microthrombosis and venous thromboembolism. However, disseminated intravascular coagulopathy (DIC)-like pictures can be seen with a bleeding tendency [2]. Some distinctions do exist between sepsis-related DIC and COVID-19-related DIC. Also, the relationship between COVID-19 infection and acute pancreatitis has been debated, as the gastrointestinal (GI) tract is a common target of COVID-19 infection and affects 25% of patients; elevated lipase is non-specific and can be seen even in the absence of clinical and imaging evidence of acute pancreatitis [3]. On the other hand, acute pancreatitis has been long known as a trigger of DIC [4].

## Case presentation

An 85-year-old male patient presented with altered mental status, which was described as being combative, for several hours before presentation. Past medical history was significant for hypertension on clonidine, labetalol, and amlodipine, hyperlipidemia on simvastatin, chronic neck pain on oxycodone, last taken the morning of his presentation, and monoclonal gammopathy of undetermined significance. He was also recently treated for meticillin-susceptible Staphylococcus aureus (MSSA) bacteremia with cefazolin. In the emergency department (ED), vitals signs included a temperature of 37.4 degrees Celsius, heart rate of 68 beats per minute, respiratory rate of 17 cycles per minute, blood pressure of 90/50 mmHg, and oxygen saturation (SpO2) of 87% requiring a 3-liter nasal cannula. On examination, he was irritable, combative, minimally responsive to verbal stimuli, and non-redirectable. Abdominal palpation revealed moderate epigastric and right upper quadrant tenderness as well as a full urinary bladder. There was evidence of dry blood at the mouth corners with scattered ecchymoses all over his body. Insertion of Foley’s catheter revealed hematuria with the initiation of continuous bladder irrigation. Blood pressure improved with a fluid challenge. 

**Figure 1:**
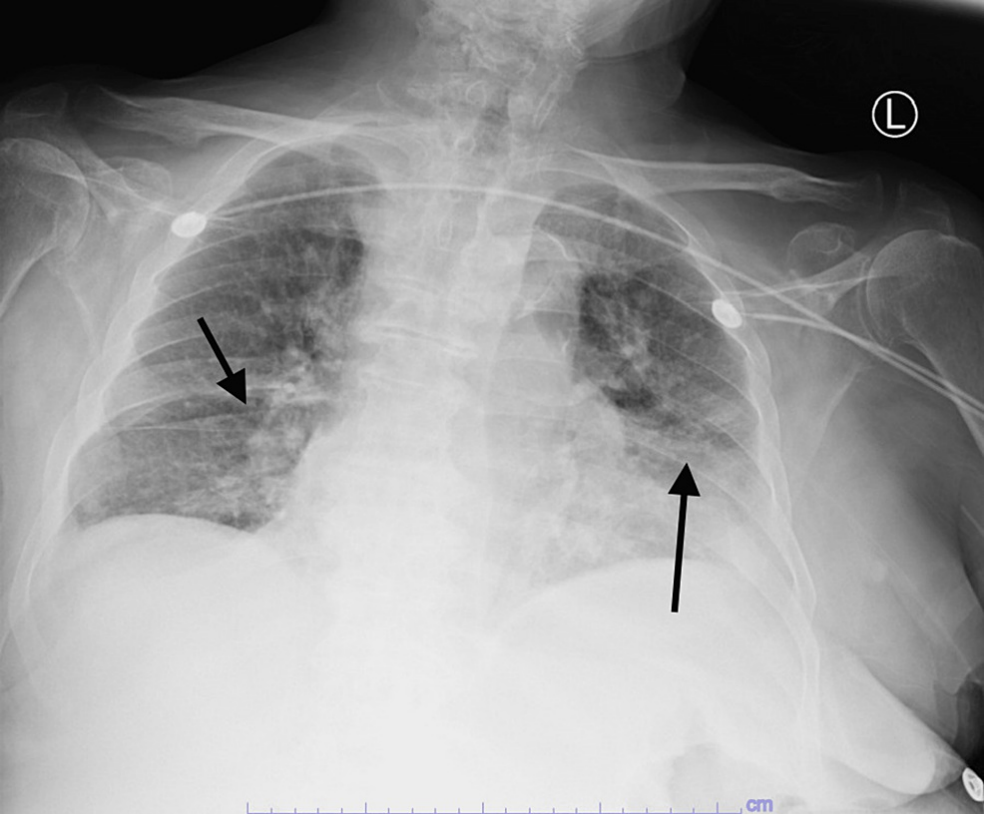
Chest X-ray Chest X-ray showing bilateral infiltrates suggestive of COVID-19 pneumonia

**Figure 2:**
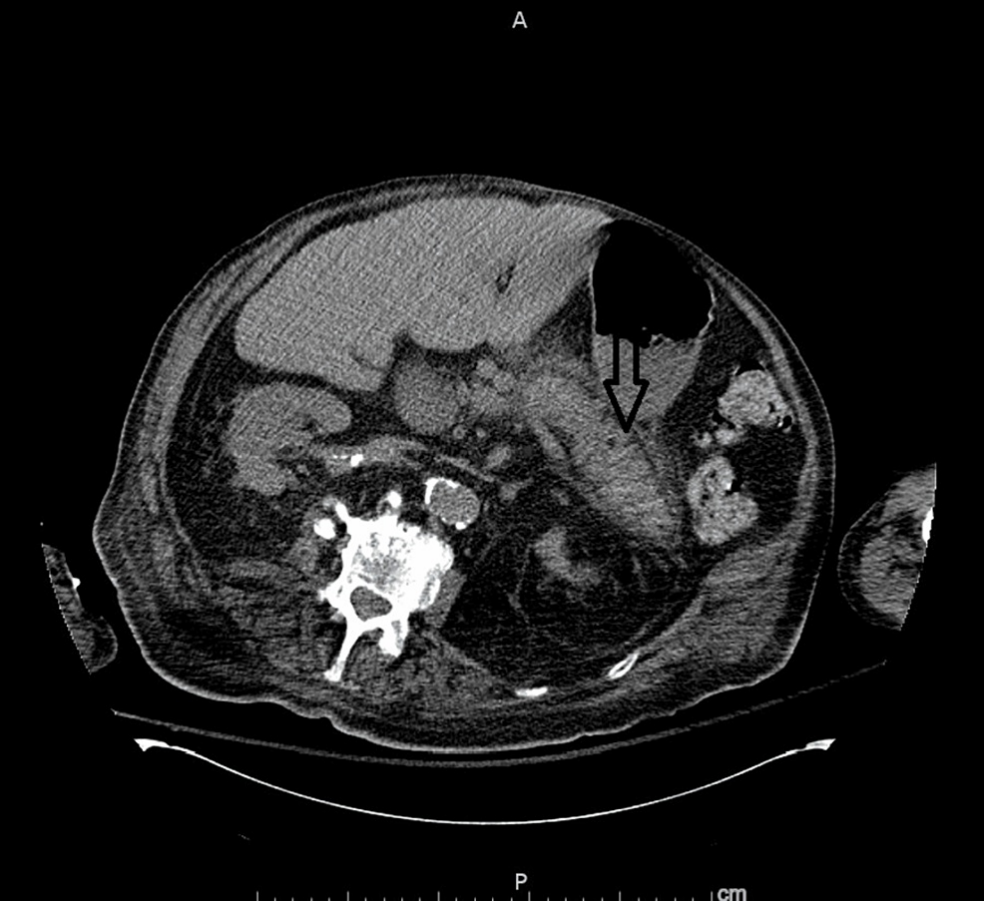
CT abdomen CT abdomen showing evidence of peripancreatic edema and stranding compatible with acute pancreatitis (arrow)

COVID polymerase chain reaction (PCR) was reactive. Chest X-ray showed bilateral diffuse opacities (Figure 1). D-dimer was elevated at 2.5 mcg/ml (reference 0-0.45 mcg/ml) with elevated C-reactive protein (CRP) at 26 mg/dl (reference less than 1 mg/dl). Ferritin was elevated at 465 ng/ml (reference 17-464 ng/ml). Computed tomography (CT) of the abdomen and pelvis without contrast showed peripancreatic edema suggestive of acute pancreatitis (Figure 2). Lipase was elevated at 17,636 U/L (reference 23-300 U/L). Urinalysis and urine drug screen were unremarkable. Basic labs showed hemoglobin of 8.3 g/dl, mean corpuscular volume (MCV) of 103 Fl, platelet count of 246,000 cells/ml, mean platelet volume of 9.7 fL (reference 9.4-12.4 fL), prolonged prothrombin time (PT) of more than 110 seconds, prolonged partial thromboplastin time (PTT) of more than 150 seconds, and international normalized ratio (INR) more than 15. These findings were double-confirmed with repeat testing. Fibrinogen level was elevated at 651 mg/dl (reference 224-476 mg/dl). The lupus anticoagulant was negative. Acute kidney injury (AKI) with elevated creatinine of 5.23 mg/dl (reference 0.66-1.25 mg/dl), elevated blood urea nitrogen (BUN) of 103 mg/dl (reference 9-20 mg/dl), with hyperkalemia of 5.9 mmol/l. The blood film showed some anisocytosis with no schistocytes.

DIC was suspected, and the patient was aggressively treated with blood product therapy, including fresh frozen plasma and cryoprecipitate, and 10 mg intravenous vitamin K therapy with gradual improvement of his coagulation profile. Remdisivir was started with no baricitinib given his active bacteremia. Dexamethasone was initiated. The lipid panel was within expected ranges and no evidence of gallstones was seen on the abdominal CT scan and ultrasound. Active hematuria and worsening mucocutaneous bleeding lead to a continuous decline in hemoglobin requiring multiple packed RBC transfusions. All cultures remained negative.

Respiratory status continued to worsen with progressive oxygen requirement to 15 liters. The renal function continued to worsen and dialysis was declined by his family. Ultimately, a goals-of-care discussion prompted the pursuit of comfort care.

## Discussion

The hypothesis that the COVID-19 virus can induce acute pancreatitis is based on the fact that acute pancreatitis can be induced by other viral infections [4]. The COVID-19 virus gains access to cells by binding to angiotensin-converting enzyme type 2 (ACE2) receptors, which are expressed in pancreatic tissue [5]. Acute pancreatitis in moderate to severe COVID-19 infection is believed to be either due to the virus itself or as part of systemic inflammation [6]. The COVID-19 virus has been isolated from pancreatic tissues at autopsies of infected patients [7]. 

Although a pancreatic injury is common among those with COVID-19, the incidence of acute pancreatitis was estimated at only 0.27% in a retrospective study involving more than 10,000 participants [8]. This same study also concluded that acute pancreatitis in the setting of COVID-19 infection carries a poor prognosis. In this case, the diagnosis of acute pancreatitis was established based on the 2012 Atlanta Classification Criteria that require two or more of the following: elevation of serum lipase more than three times the upper normal level, imaging evidence of pancreatitis, and typical exam finding of epigastric pain and tenderness. Our patient had all three criteria [9]. Although elevated lipase is nonspecific and can be seen in COVID-19 infection without acute pancreatitis due to impaired pancreatic microcirculation, increased intestinal permeability, renal impairment, diarrhea, diabetes, critical illness in the ICU or opioid use, our patient had imaging and clinical evidence of acute pancreatitis [10]. In this case, typical risk factors of acute pancreatitis of alcoholism, gallstones, medications, and hyperlipidemia were ruled out. Thus, it is possible to hypothesize that acute pancreatitis was induced by COVID-19 infection, although a causal relationship is difficult to ascertain.

A large prospective study concluded that the incidence of DIC among COVID-19 survivors was 0.6% and 71% among non-survivors [11]. COVID-19-associated coagulopathy mimics other infections-associated coagulopathies, especially DIC [12]. It was referred to by some authors as a DIC-like state that fulfills the International Society on Thrombosis and Haemostasis (ISTH) diagnostic criteria of DIC [13]. However, some distinctions exist. Fibrinogen level is usually elevated in COVID-19 patients, likely as an acute phase response [11]. Platelet count is usually normal or even elevated [14].

Unlike sepsis-associated-DIC, which is usually of suppressed fibrinogen type with mild elevation of both fibrinogen degradation products and D-dimer with more pronounced thrombocytopenia, COVID-19-associated coagulopathy is associated with less thrombocytopenia [2]. In the same study mentioned previously, it was noted that DIC in COVID-19 patients was of an enhanced fibrinolytic type with mild elevation of D-dimer and more pronounced elevation of fibrinogen degradation products (FDP). Change from enhanced fibrinolytic type DIC to suppressed fibrinolytic type DIC can also happen and is evidenced by a sharp decline in fibrinogen level [1]. We cannot comment on the type of DIC in our patient, as FDP was not measured.

COVID-19-induced coagulopathy usually mimics chronic DIC where thrombosis predominates rather than acute DIC where bleeding predominates [13]. In our patient, the ISTH criteria were met, with 2 points for severely prolonged PT and 3 points for severely elevated D-dimer in the setting of severe infection and bleeding was clinically evident thus establishing the diagnosis of DIC. Whether or not COVID-19 was the only inciting factor of DIC is difficult to ascertain.

DIC could also have been precipitated by acute pancreatitis, as it has been reported in acute severe pancreatitis, where it has prognostic significance [15]. DIC is a risk factor for multiple organ failure in acute severe pancreatitis [16]. Multiple factors contribute to DIC in acute severe pancreatitis, including systemic inflammation, endothelial injury, and direct coagulation activation [3].

## Conclusions

COVID-19 coagulopathy can present with a DIC-like picture with some distinctions, including elevated fibrinogen and normal or elevated platelet count. Although it has been reported to mimic chronic DIC with predominant thrombosis, an acute DIC picture with predominant bleeding can also be seen. COVID-19 is also a plausible risk factor for acute pancreatitis.
